# Supplementary material for: A protein adaptor mediating Ap4A-dependent control of protein acetylation
Source: Nat Commun. 2026 Feb 23;17:3089. doi: 10.1038/s41467-026-70006-0 (PMC13039410; doi:10.1038/s41467-026-70006-0)
Supplement: Supplementary file 1 — Supplementary Information [file 41467_2026_70006_MOESM1_ESM.pdf]

Supplementary information for:

A protein adaptor mediating Ap4A-dependent control of protein acetylation.

Liujuan Zheng[1,2], Megan K. M. Young[3], Wieland Steinchen[2], Zhiyong Guo[4], Ekaterina Jalomo-Khayrova[2], Bobby Xuanyu Liu[3], Fabiana Burchert[2], Patricia Bedrunka[2], Christopher-Nils Mais[2], Jan Pane-Farre[2], Mathias Girbig[1], Uwe Linne[2], Aude Trinquier[3], Aitao Li[4], Georg Hochberg[1], Johannes Freitag[2], Jue D. Wang[3,5] and Gert Bange[1,2,5]

[1]Max-Planck Institute for Terrestrial Microbiology, Karl-von-Frisch Strasse 14, 35043 Marburg, Germany
[2]Marburg University, Center for Synthetic Microbiology (SYNMIKRO) & Departments of Chemistry, Karl-von-Frisch Strasse 14, 35043 Marburg, Germany
[3]Department of Bacteriology, University of Wisconsin-Madison, Madison, WI, USA
[4]State Key Laboratory of Biocatalysis and Enzyme Engineering, Hubei Key Laboratory of Industrial Biotechnology, School of Life Sciences, Hubei University, Wuhan 430062, China
[5]Corresponding authors: gert.bange@synmikro.uni-marburg.de and wang@bact.wisc.edu

The file contains:
Supplementary Figures 1-23
Supplementary Tables 1-6

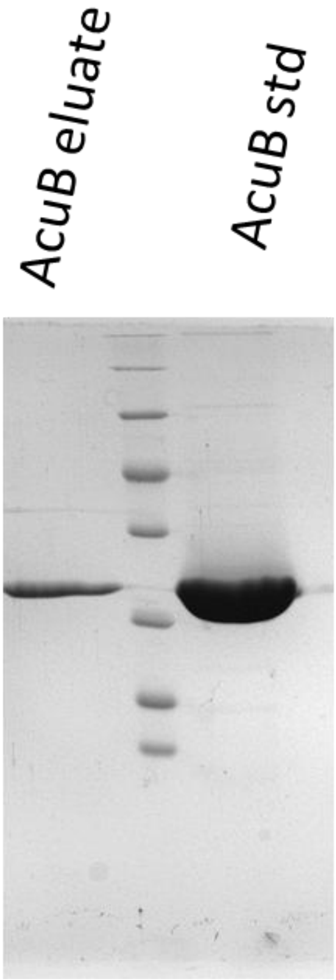

Supplementary Fig. 1. In vivo pulldown of AcuB in *Bacillus subtilis*. Coomassie-stained SDS-PAGE of (*Bs*)AcuB-Strep overexpressed in *B. subtilis* and purified with MagStrep "type3" XT beads**.** Mass spectrometry data of the empty vector control and the AcuB pulldown eluate in *B. subtilis* are provided in Supplementary data 1 and Supplementary data 2. Pulldown experiments were performed twice with similar results, and one representative result is shown. Source data are provided as a Source Data file.

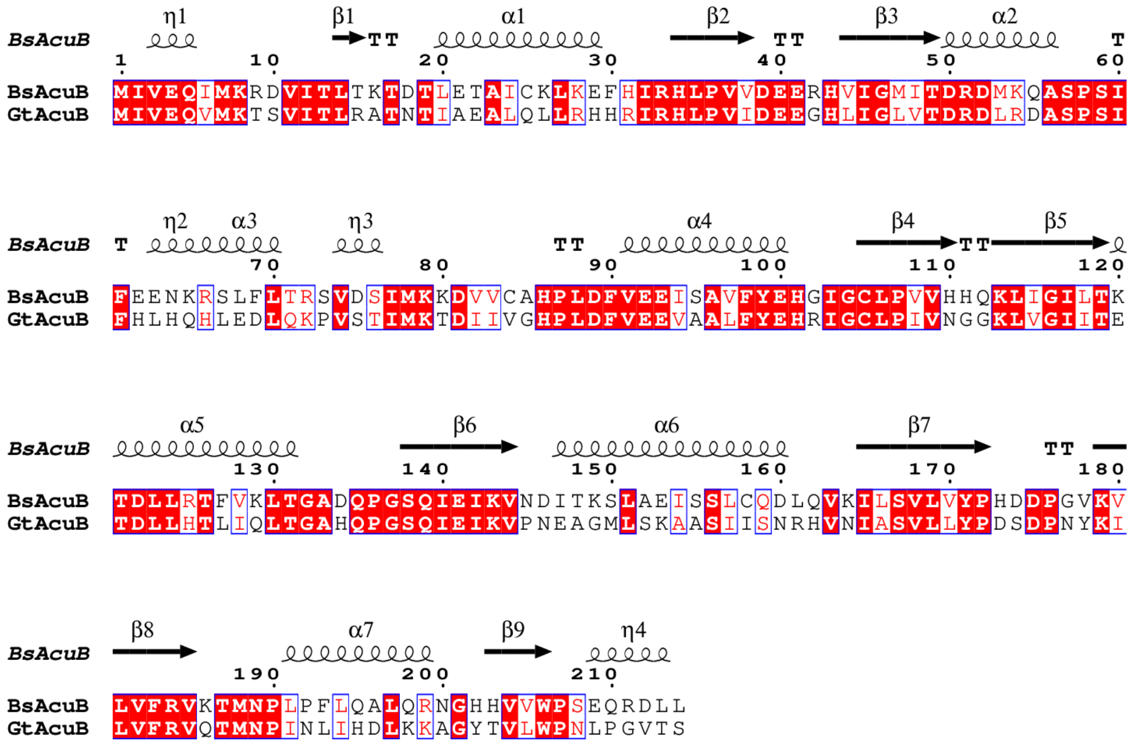

Supplementary Fig. 2. Sequence alignment of (*Bs*)AcuB and (*Gt*)AcuB. (*Bs*)AcuB and (*Gt*)AcuB show a 53.85% sequence identity and a highly similar domain organization.

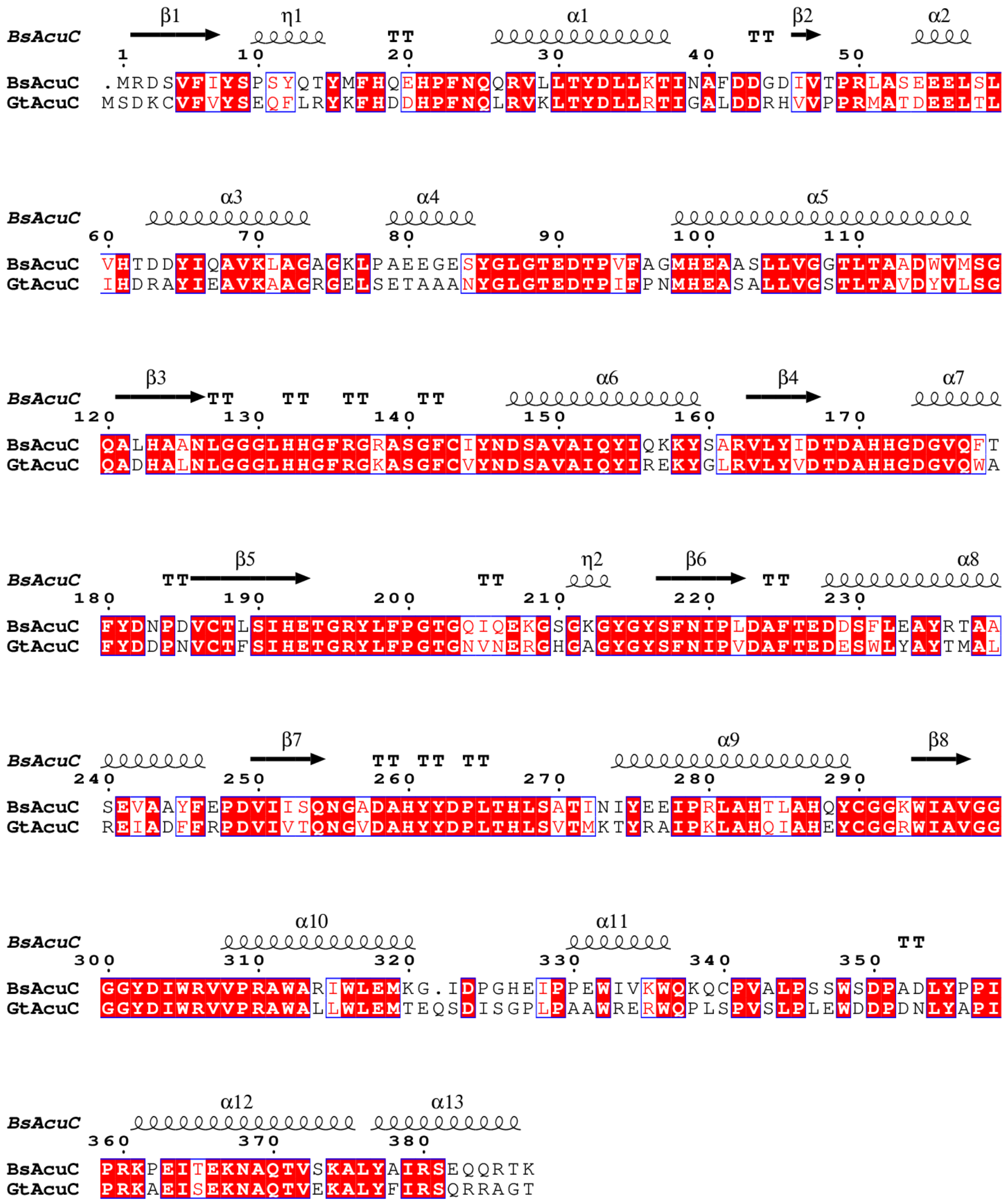

Supplementary Fig. 3. Sequence alignment of (*Bs*)AcuC and (*Gt*)AcuC. (*Bs*)AcuC and (*Gt*)AcuC show a 65.37% sequence identity.

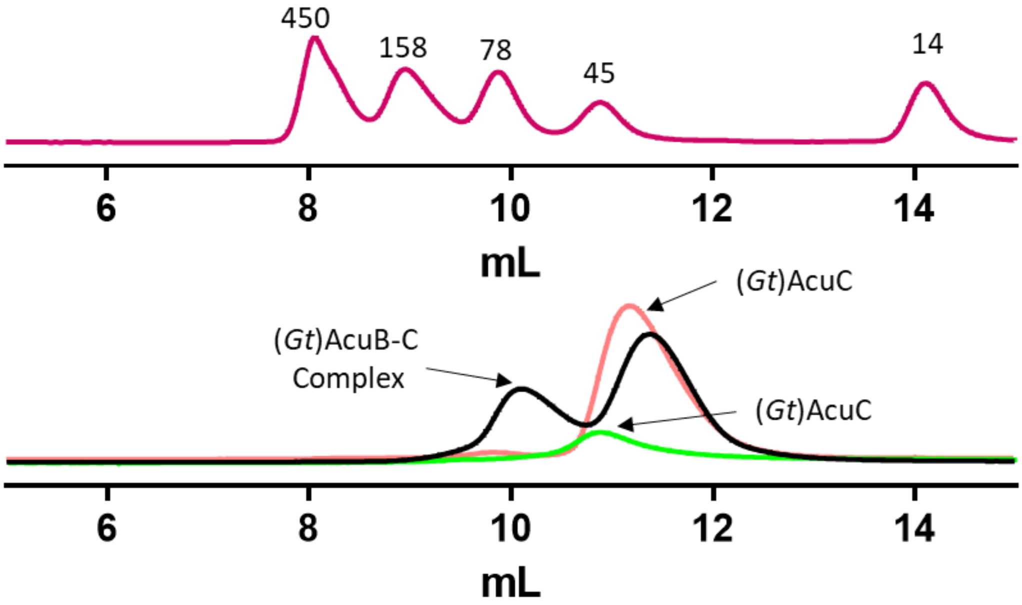

Supplementary Fig. 4. Analytical size-exclusion chromatography (SEC) profiles of (*Gt*)AcuB, (*Gt*)AcuC, and their complex. Chromatograms show the elution profiles of (*Gt*)AcuB (green), (*Gt*)AcuC (orange), and the (*Gt*)AcuB–(*Gt*)AcuC complex (black), alongside molecular weight standards: ferritin (440 kDa), aldolase (158 kDa), conalbumin (78 kDa), ovalbumin (45 kDa), and RNase A (14 kDa). All experiments were performed at least twice with similar results, and one representative result is shown. Source data are provided as a Source Data file.

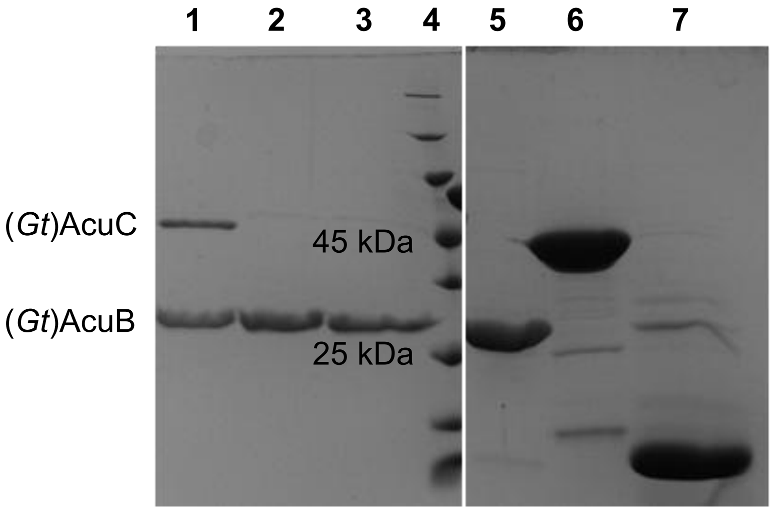

Supplementary Fig. 5. Pulldown of AcuB with YabA. The pulldown assay was performed as described in the Methods section. (*Gt*)AcuB (N-terminal Strep-tag and C-terminal His-tag) was used as the bait, while N-His-tagged (*Gt*)AcuC (**lane 1**) and (*Bs*)YabA, without (**lane 2**) and with Ap4A (**lane 3**), served as preys. Strep-Tactin®-coated magnetic beads (IBA) were used for the pulldown experiments. Interactions between (*Gt*)AcuB and (*Gt*)AcuC served as a positive control (**lane 1**). However, no binding was detected between (*Gt*)AcuB and (*Bs*)YabA under the tested conditions. **Lane 4** shows the size standard. **Lane 5 – 7** show the input controls ordered as follows: (*Gt*)AcuB, (*Gt*)AcuC and (*Bs*)YabA. All experiments were performed at least twice with similar results, and one representative graph is shown here. Source data are provided as a Source Data file.

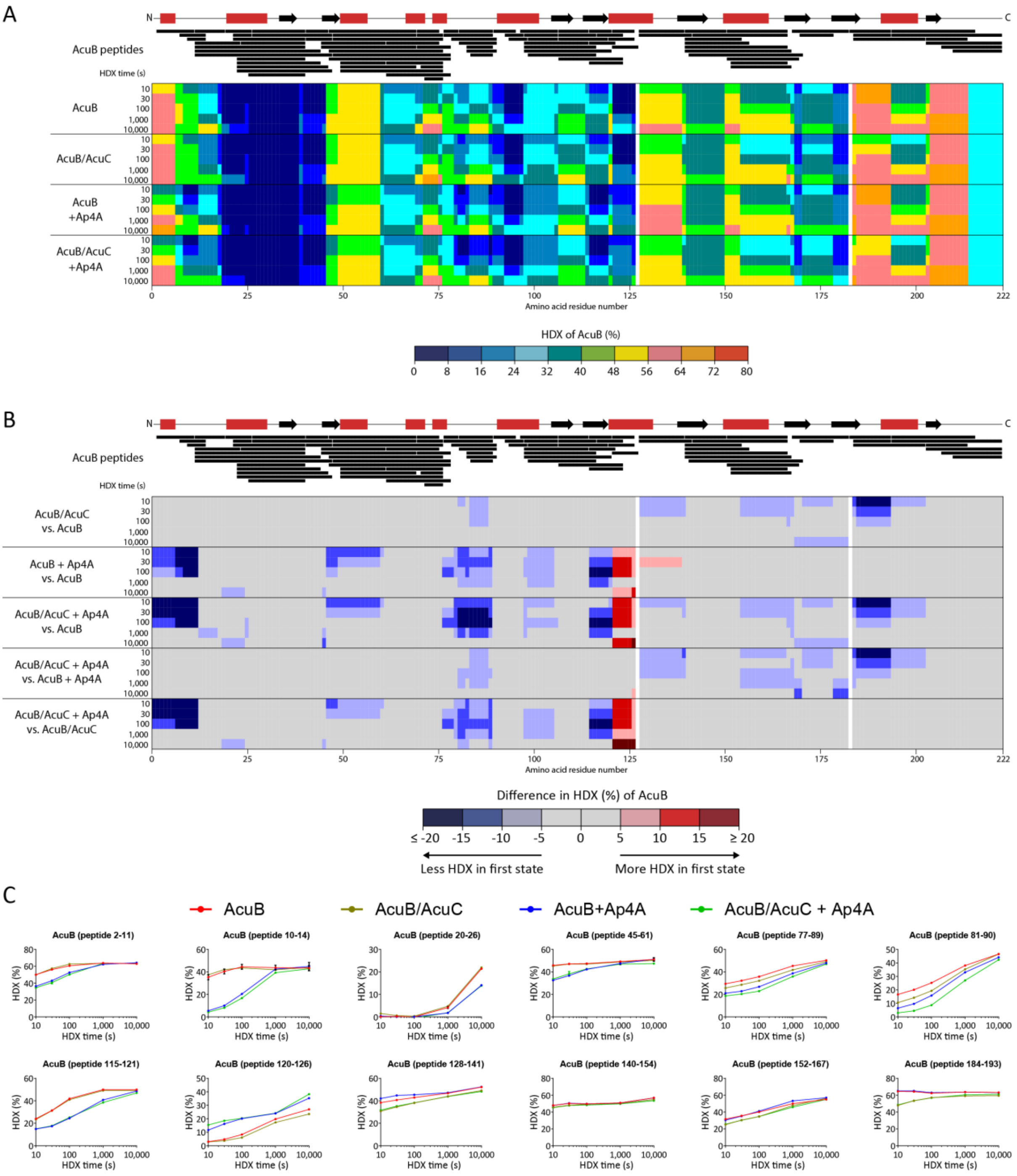

Supplementary Fig. 6. Interface of (*Gt*)AcuB in (*Gt*)AcuB-AcuC, (*Gt*)AcuB-Ap4A, and Ap4A-(*Gt*)AcuB-AcuC complexes as revealed by HDX-MS analysis. A. Each black bar denotes a (*Gt*)AcuB peptide identified by HDX-MS as well as a residue-specific HDX of (*Gt*)AcuB, either in isolation or in context of the complexes. **B.** The difference in residue-specific HDX of (*Gt*)AcuB when bound in the complexes versus (*Gt*)AcuB alone. **C.** HDX of selected representative (*Gt*)AcuB peptides. Data represent mean ± s.d. of n = 3 technical replicates (individual HDX reactions). Source data are provided as a Source Data file.

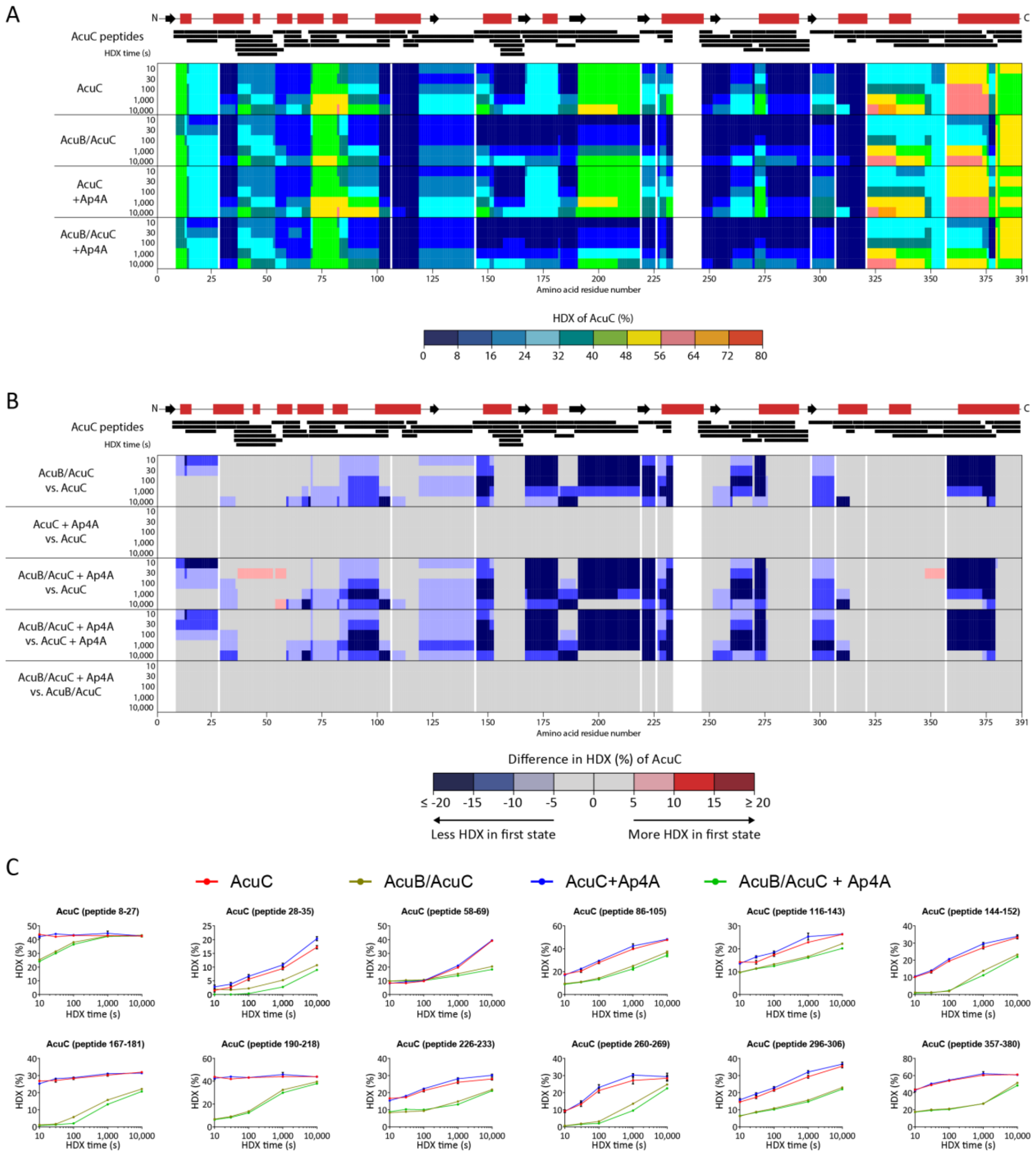

Supplementary Fig. 7. Interface of (*Gt*)AcuC in (*Gt*)AcuB-AcuC, (*Gt*)AcuB-Ap4A, and Ap4A-(*Gt*)AcuB-AcuC complexes as revealed by HDX-MS analysis. A. Each black bar denotes a (*Gt*)AcuC peptide identified by HDX-MS as well as residue-specific HDX of (*Gt*)AcuC, either in isolation or in context of the complexes. **B.** The difference in residue-specific HDX of (*Gt*)AcuC when bound in the complexes versus (*Gt*)AcuC alone. **C.** HDX of selected representative (*Gt*)AcuC peptides. Data represent mean ± s.d. of n = 3 technical replicates (individual HDX reactions). Source data are provided as a Source Data file.

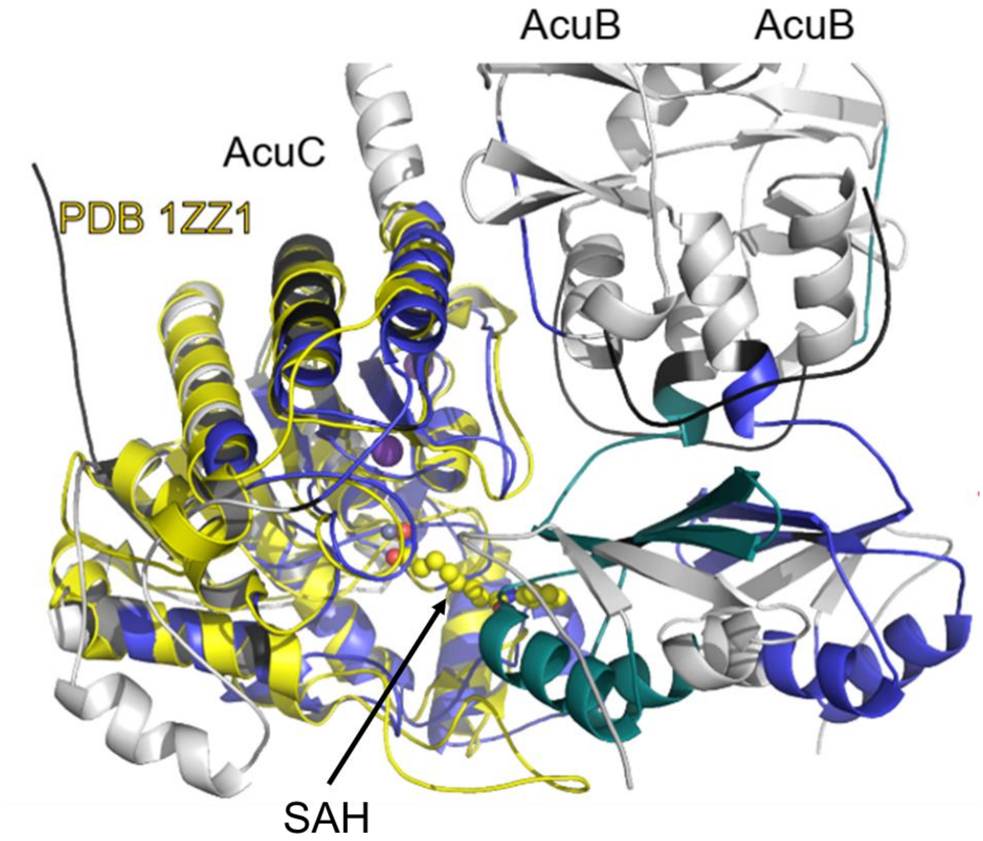

Supplementary Fig. 8. Alignment of the deacetylase (yellow) bound to suberoylanilide hydroxamic acid (SAHA) from *Alcaligenaceae bacterium* (PDB ID: 1ZZ1) with the (Gt)AcuB–AcuC complex. The ACT domain in AcuB binds in a manner similar to SAHA, blocking the substrate entry site and interacting with the catalytic center of AcuC.

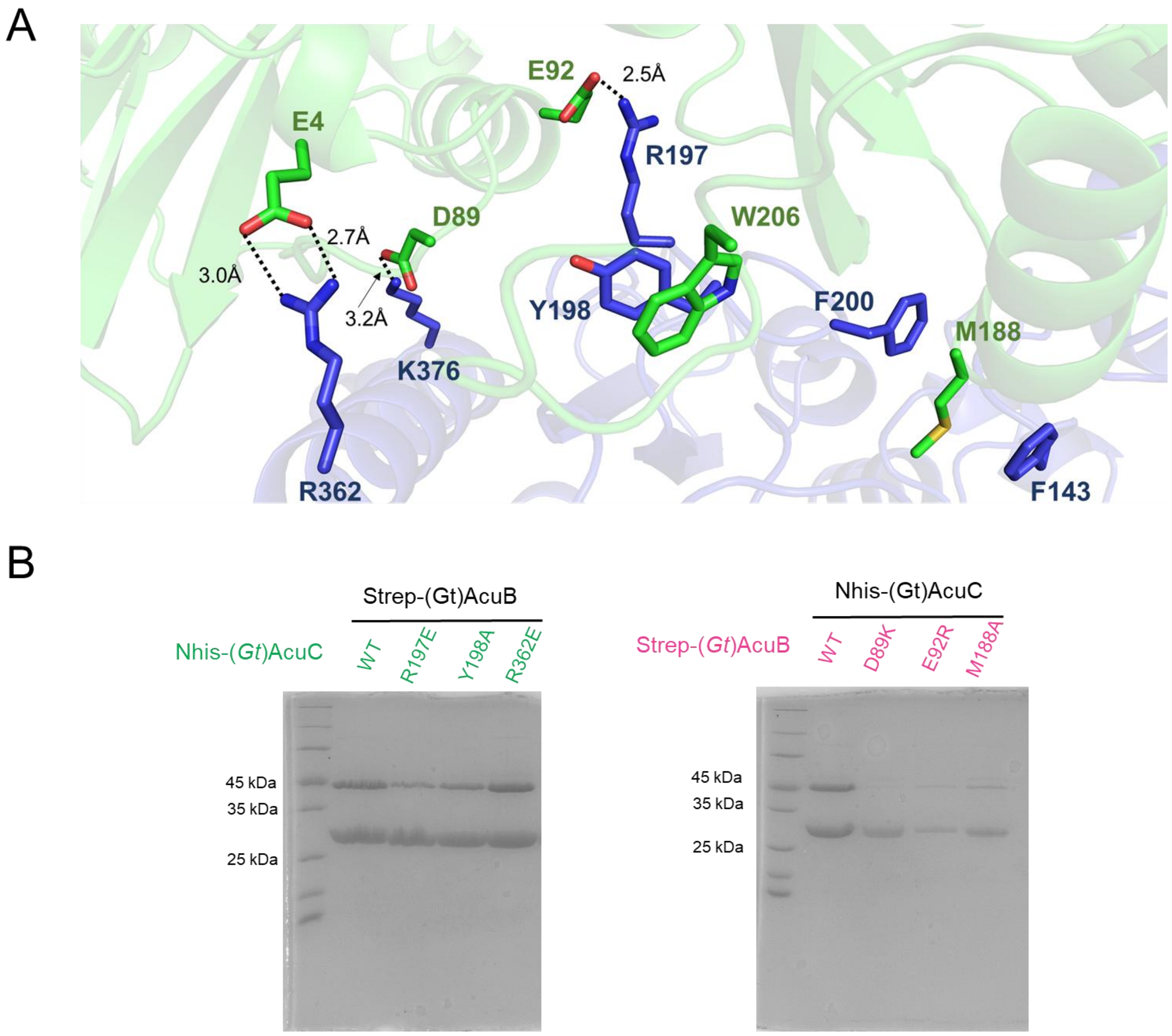

Supplementary Fig. 9. Identification and validation of interfacial residues mediating the (*Gt*)AcuB–AcuC interaction. A. Structural analysis of the (*Gt*)AcuB–AcuC complex reveals that the interface is stabilized by multiple interactions, including salt bridges between (*Gt*)AcuB_E4–(*Gt*)AcuC_R362, (*Gt*)AcuB_D89–(*Gt*)AcuC_K378, and (*Gt*)AcuB_E92–(*Gt*)AcuC_R197, as well as π–π and hydrophobic contacts between (*Gt*)AcuB_W206–(*Gt*)AcuC_Y197 and (*Gt*)AcuB_M188–(*Gt*)AcuC_F200/F143. **B**. To verify these predicted contacts, single-point mutants (*Gt*)AcuB_D89K, E92R, M188A and (*Gt*)AcuC_R197E, Y198A, R362E were generated and analyzed by pulldown assays. Mutations D89K, E92R, and M188A in AcuB markedly disrupted complex formation, while R197E and Y198A in AcuC moderately weakened the interaction. In contrast, R362E had little effect. These results confirm the key interfacial residues responsible for (*Gt*)AcuB–AcuC complex formation. Source data are provided as a Source Data file.

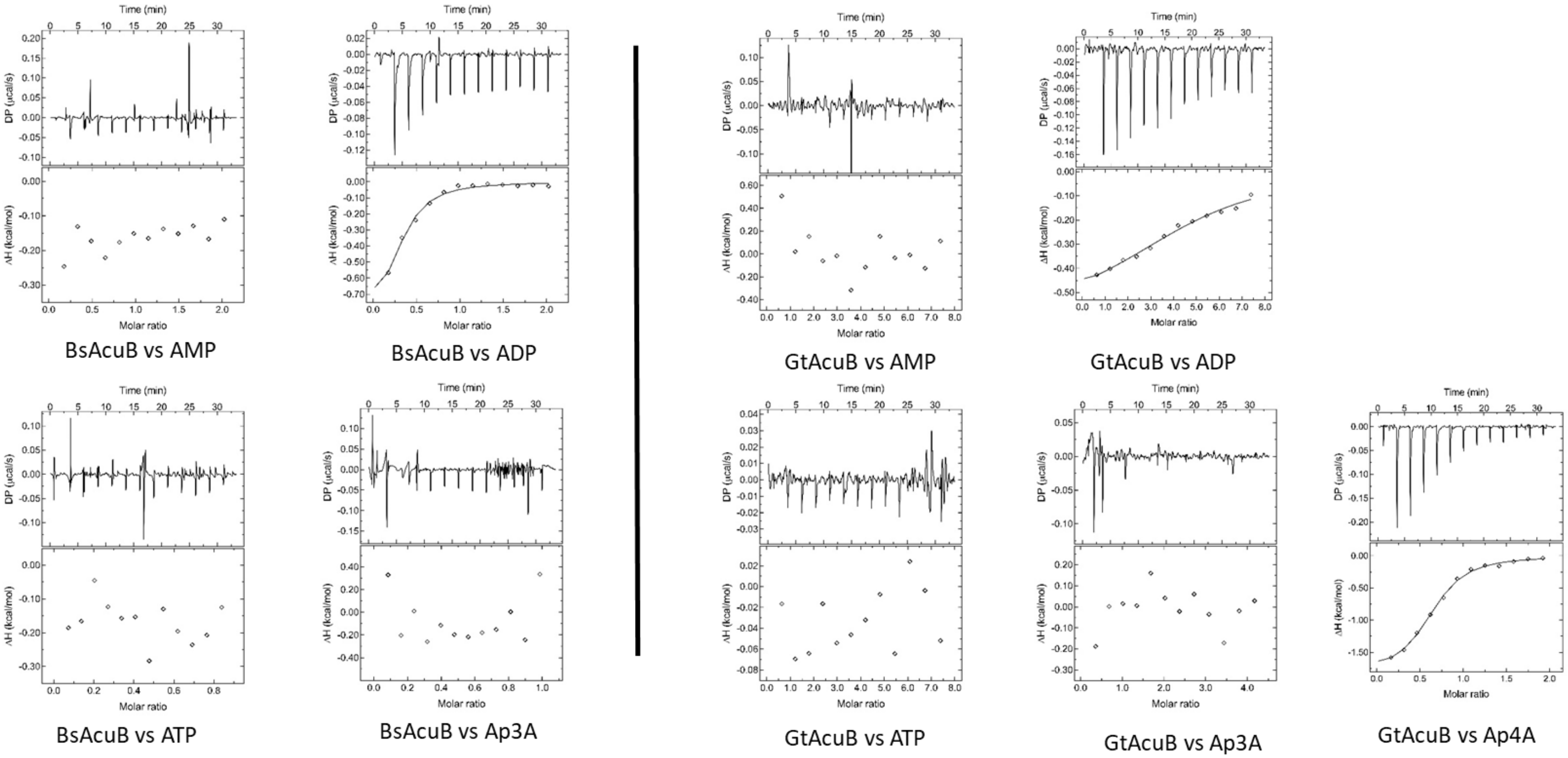

Supplementary Fig. 10. ITC measurements of AcuB with different nucleotides. ITC was performed at 25 °C using a MicroCal PEAQ-ITC (Malvern Panalytical). Proteins ((*Bs*)AcuB or (*Gt*)AcuB, 40–120 µM) and nucleotides (0.4–1 mM, ⩾95% purity, Jena Bioscience) were prepared in 20 mM HEPES, 20 mM $MgCl_2$, 20 mM KCl, 200 mM NaCl, pH 7.5. Titrations involved 13 injections (0.4 µl first, then 3 µl each) with 150 s intervals. Data were analyzed using the “one binding site” model in MicroCal PEAQ-ITC Analysis Software and plotted with GraphPad Prism. Ligands included AMP, ADP (very weak, no reliable K_D), ATP, Ap3A.

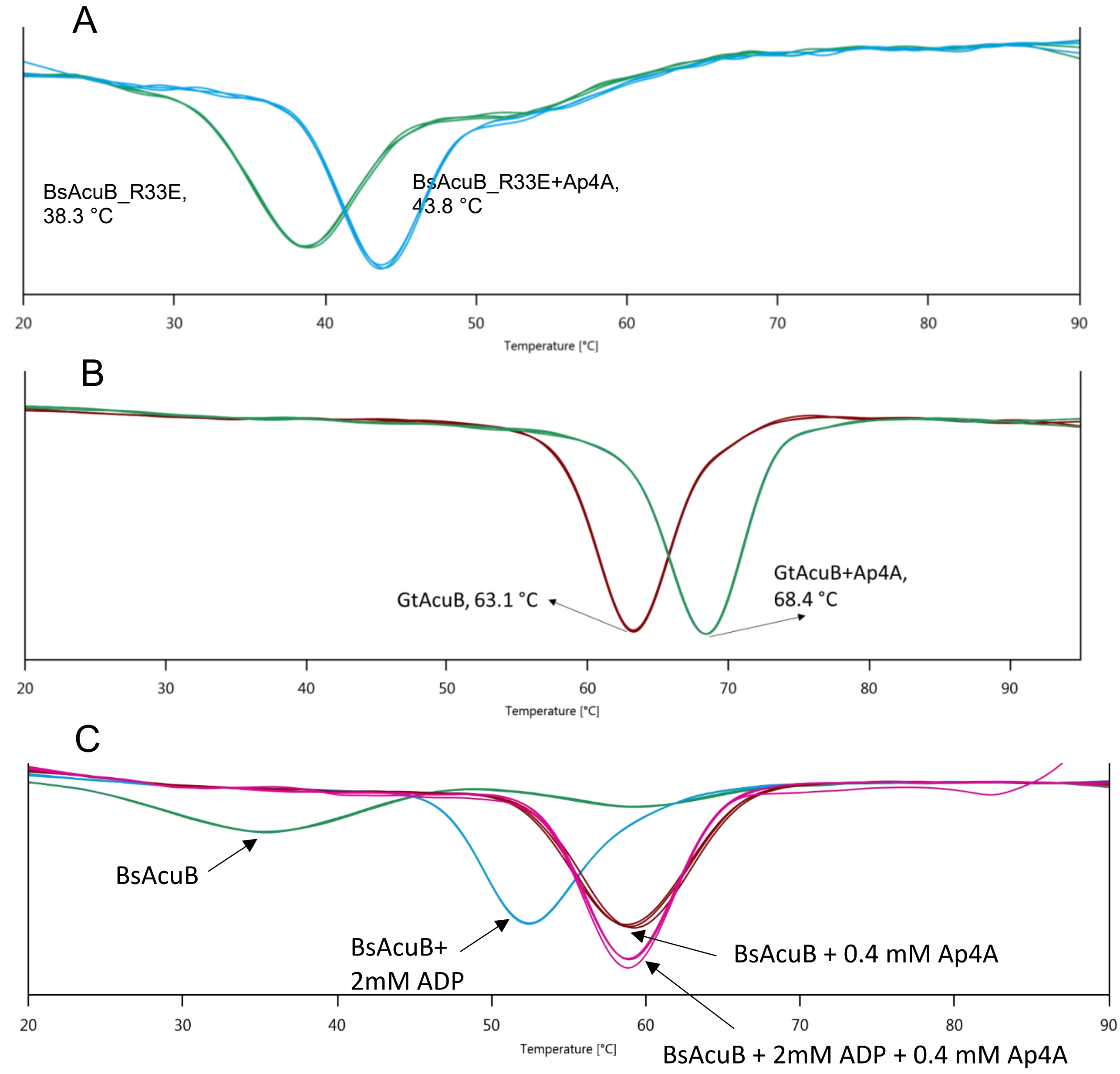

Supplementary Fig. 11. Thermostability of AcuB and its mutant with and without Ap4A. A. Thermal stability of (*Bs*)AcuB_R33E with and without Ap4A. **B**. Thermal stability of (*Gt*)AcuB with and without Ap4A. Thermal stability was assessed using NanoDSF on a Prometheus NT.48 (NanoTemper Technologies). **C**. Competition thermoshift assay showing that Ap4A preferentially binds to (*Bs*)AcuB even in the presence of excess ADP. When 2 mM ADP was added at a fivefold higher concentration than Ap4A (0.5 mM), the thermal shift remained comparable to that induced by Ap4A alone, indicating that Ap4A is the dominant and higher-affinity ligand for (*Bs*)AcuB. Proteins (1 mg/mL in SEC buffer with different ligands) were loaded into UV-transparent capillaries (2 µL each). Samples were heated from 20 °C to 95 °C at 1 °C/min, monitoring intrinsic fluorescence at 330 nm and 350 nm. The melting temperature (Tm) was determined from the 350/330 nm fluorescence ratio using PR.ThermControl software. Each experiment was performed in triplicates (n = 3). Source data are provided as a Source Data file

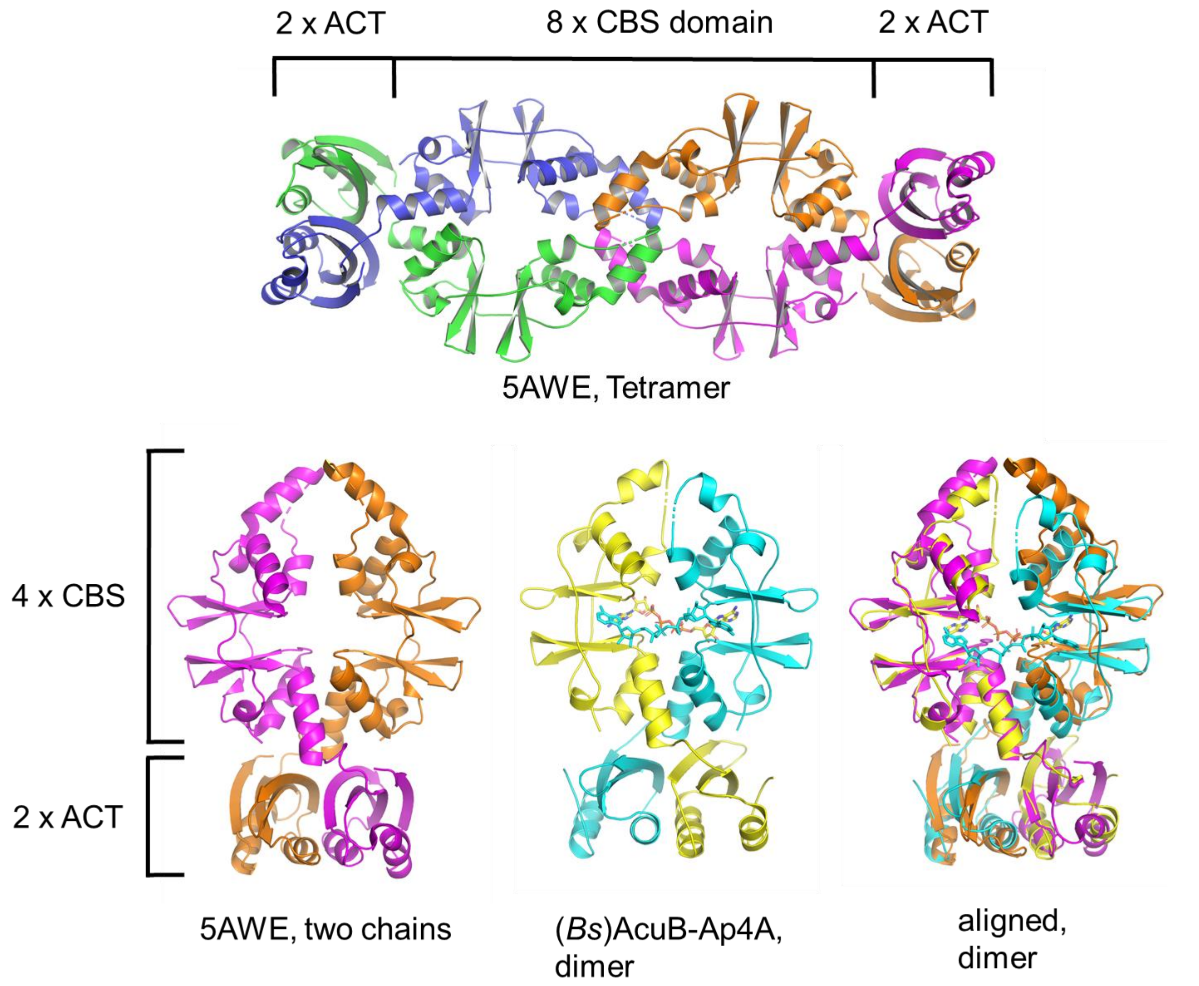

Supplementary Fig. 12. Crystal structure of AcuB homolog 5AWE from *Thermus thermophilus*[1]. The structure 5AWE is as a tetramer (dimer-of-dimers), while (*Bs*)AcuB–Ap4A forms a dimer. Superposition of 5AWE and (*Bs*)AcuB results in a good alignment with an RMSD of 2.319 Å.

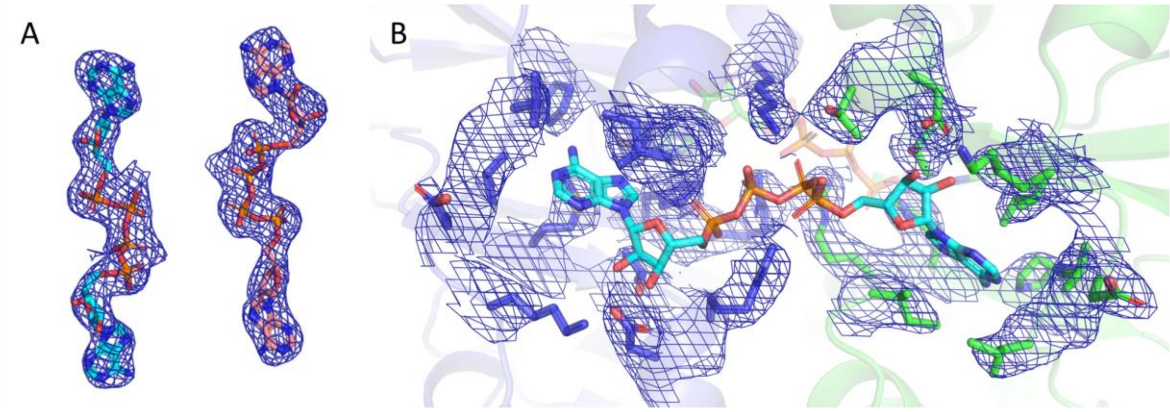

Supplementary Fig. 13. Electron density map showing the coordination of Ap4A-1 and Ap4A-2 within the CBS domains of (*Bs*)AcuB. A The mF_o–D F_c electron density map (blue mesh), contoured at 3.0 σ, shows two Ap4A molecules (left and right). **B** The mF_o–D F_c omit map (calculated after removal of the ligand, blue mesh), contoured at 1.0 σ, highlights the electron density around Ap4A.

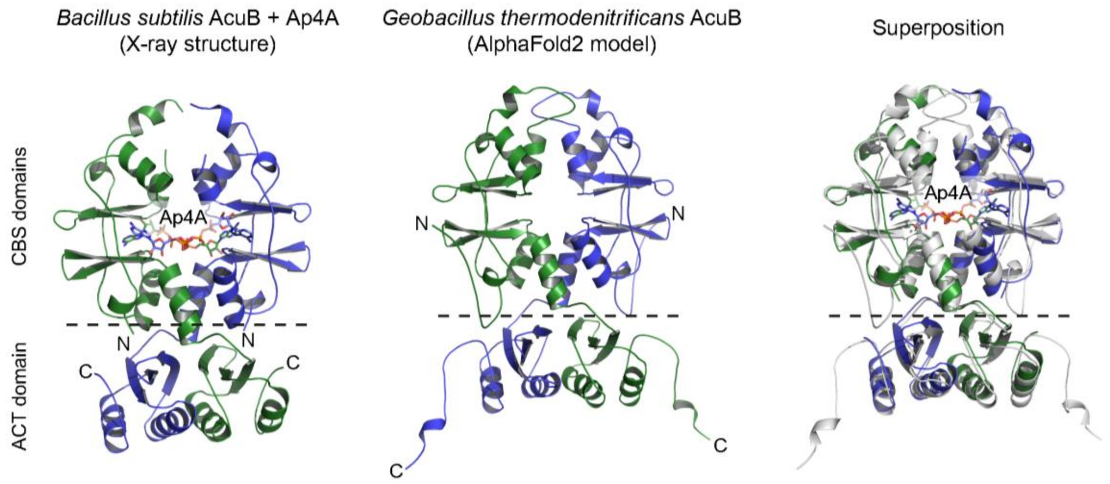

Supplementary Fig. 14. Superposition of (*Bs*)AcuB crystal structure binding with Ap4A to (*Gt*)AcuB AlphaFold model. The crystal structure of AcuB from *Bacillus subtilis* bound to Ap4A (left), the AlphaFold2 model)[2] of AcuB from *Geobacillus thermodenitrificans* (middle) and their superposition (right) are shown in cartoon representation. Two Ap4A molecules identified in the *B. subtilis* AcuB crystal structure are shown as sticks and colored per atom.

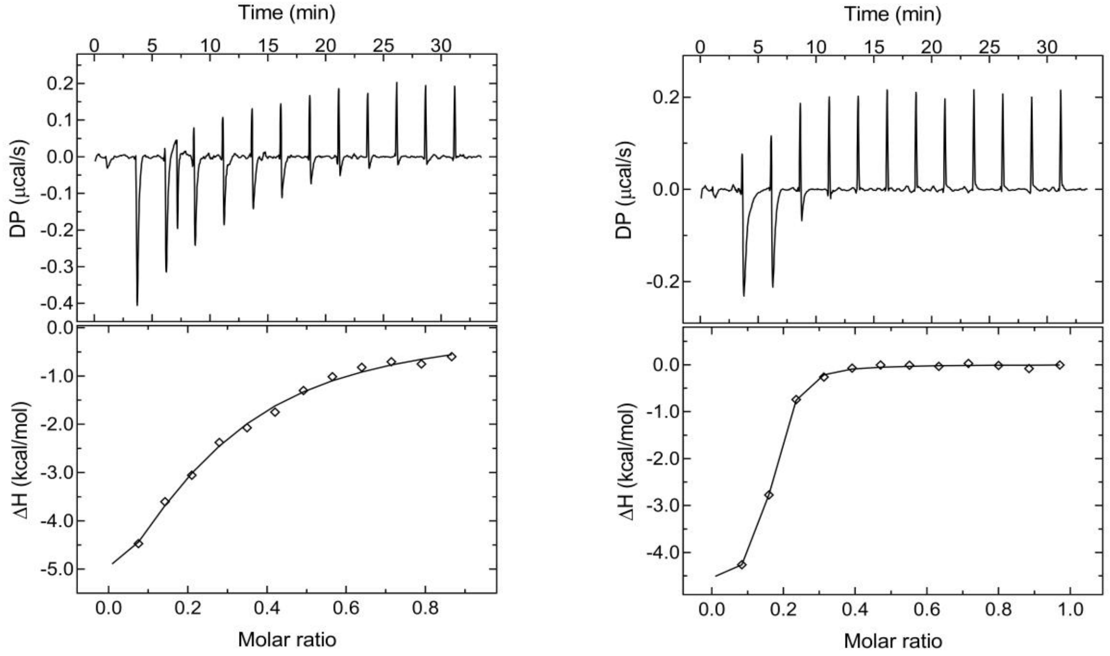

R51E vs Ap4A D122A vs Ap4A

Supplementary Fig. 15. ITC measurement of (*Bs*)AcuB_mutants with Ap4A. ITC was performed as described in Supplementary Fig. 10. Proteins (*Bs*)AcuB_R51E or (*Bs*)AcuB_D122A (40–120 µM) and nucleotides (0.4–1 mM, ⩾95% purity, Jena Bioscience) were prepared in 20 mM HEPES, 20 mM $MgCl_2$, 20 mM KCl, and 200 mM NaCl, pH 7.5. Titrations consisted of 13 injections (0.4 µL followed by 3 µL each) with 150 s intervals. Data were analyzed using the "one binding site" model in the MicroCal PEAQ-ITC Analysis Software and plotted with GraphPad Prism. The binding affinities (Kd) for (*Bs*)AcuB_R51E and (*Bs*)AcuB_D122A toward to Ap4A were 28.6 ± 15.0 µM and 0.72 ± 0.08 µM, respectively.

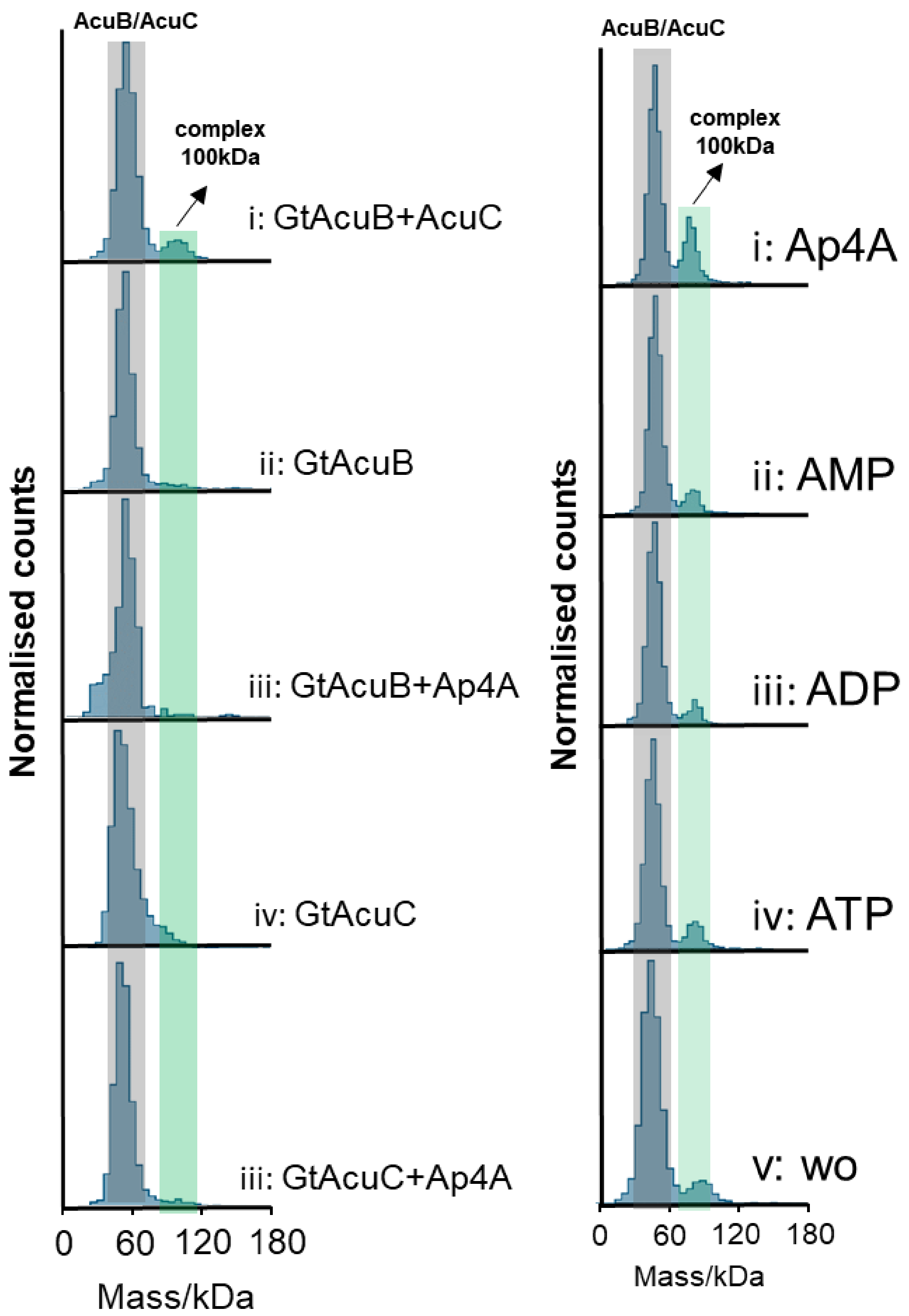

Supplementary Fig. 16. Influence of different nucleotides to the (*Gt*)AcuB-AcuC complex. The peak around 50 kDa corresponds to either the (*Gt*)AcuB dimer or the AcuC monomer (*left*), while the peak around 100 kDa represents the (*Gt*)AcuB–AcuC complex (*right*). For mass photometry (MP) analysis, 500 nM (*Gt*)AcuB, 500 nM (*Gt*)AcuC, and 1 mM Ap4A were premixed, resulting in final protein concentrations of 25 nM each and a final Ap4A concentration of 50 µM. Under these conditions, the intensity of the complex peak (~100 kDa) was detected, each experiment was performed in triplicates (n = 3), and a representative spectra is shown here. Source data are provided as a Source Data file.

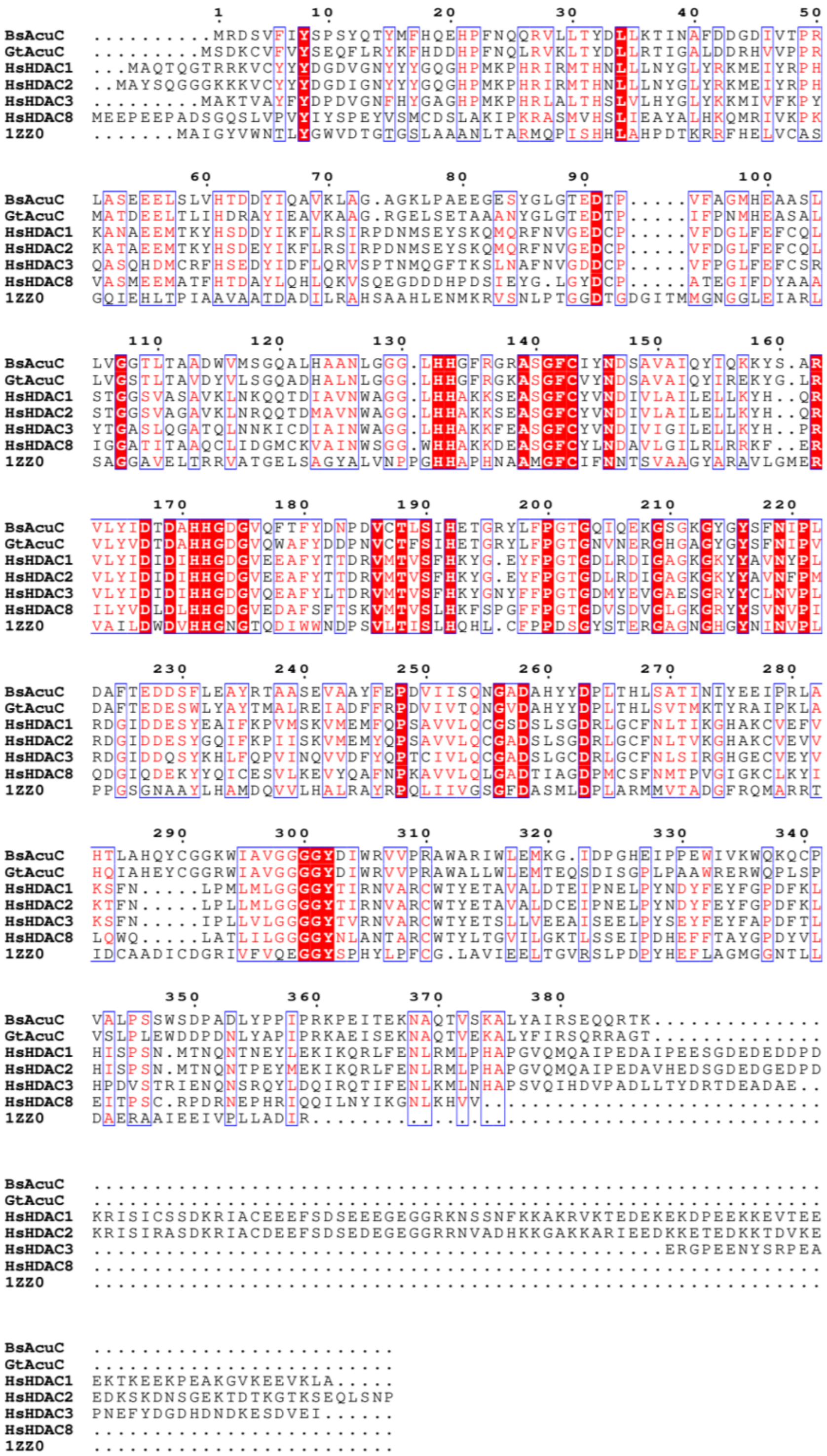

Supplementary Fig. 17. Sequence alignment of AcuCs to the known *Hs*HDACs and KDAC. The key catalytic residues including Asp259, Asp170, His172, Tyr303, His133, and His134 are conserved.

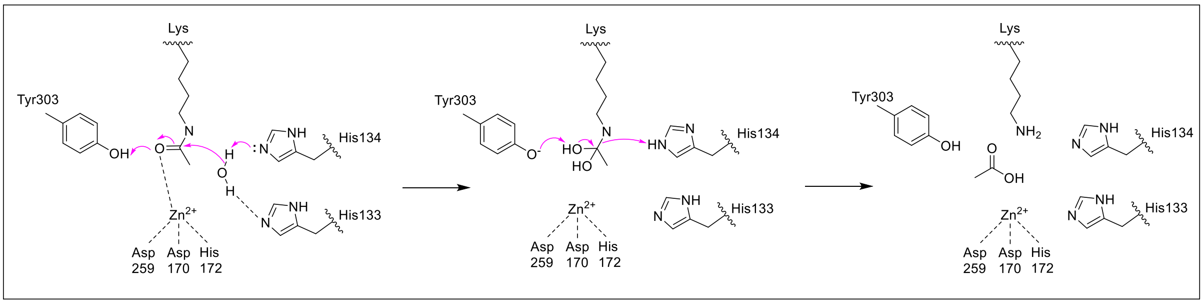

Supplementary Fig. 18. Potential mechanism of Class I HDAC activity for AcuC[3,4]. Analogous to zinc proteases, the carbonyl oxygen of the N-acetyl amide coordinates with the zinc ion, positioning the carbonyl carbon adjacent to an activated water molecule. The zinc ion polarizes the carbonyl group, increasing the electrophilicity of the carbon and properly orienting the water for nucleophilic attack. The nucleophilicity of the water is further enhanced by the nearby buried residue His134. Nucleophilic attack by the water on the carbonyl carbon generates a tetrahedral oxyanion intermediate, which is stabilized by zinc coordination and possibly a hydrogen bond from Tyr303. In the final step, the carbon–nitrogen bond is cleaved, and the departing amine group accepts a proton from His134, resulting in the release of acetate and lysine.

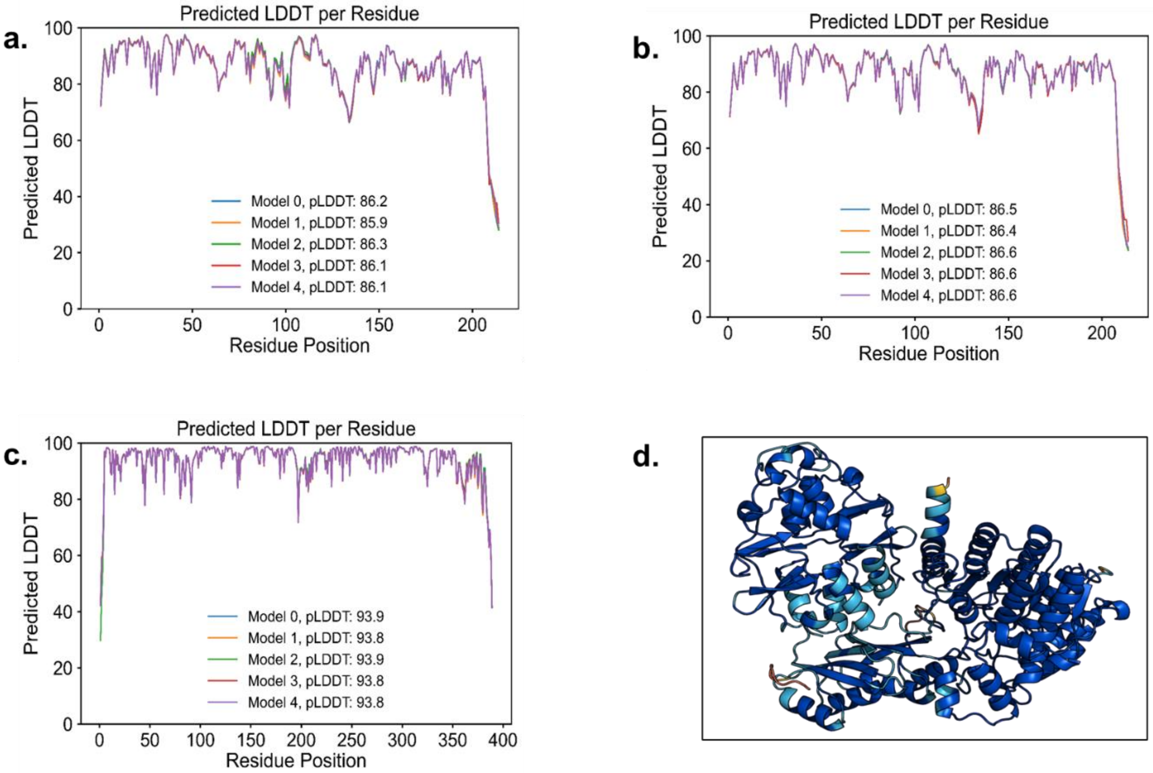

Supplementary Fig. 19. Structural confidence and overall quality of the AlphaFold-3 model for the 2AcuB_1AcuC trimer. A–C. Per-residue predicted Local Distance Difference Test (pLDDT) scores for the five AlphaFold-3–predicted models of the 2AcuB_1AcuC trimer. Panels show the pLDDT profiles for (a) AcuB-A chain, (b) AcuB-B chain, and (c) AcuC. Each line represents one of the five independent AF3 predictions, illustrating the high and consistent confidence across all models. Most residues exhibit pLDDT > 90 (very high confidence), while nearly all remaining regions show pLDDT > 70 (confident), indicating overall structural reliability. **D.** Three-dimensional representation of the top-ranked AF3 model of the 2AcuB_1AcuC trimer, colored by pLDDT value: blue (pLDDT > 90, very high confidence), cyan (70 < pLDDT ≤ 90, confident), yellow (50 < pLDDT ≤ 70, low confidence), and orange/red (pLDDT ≤ 50, very low confidence). The structure demonstrates uniformly high confidence throughout both AcuB subunits and AcuC, supporting the reliability of the predicted trimer interface.

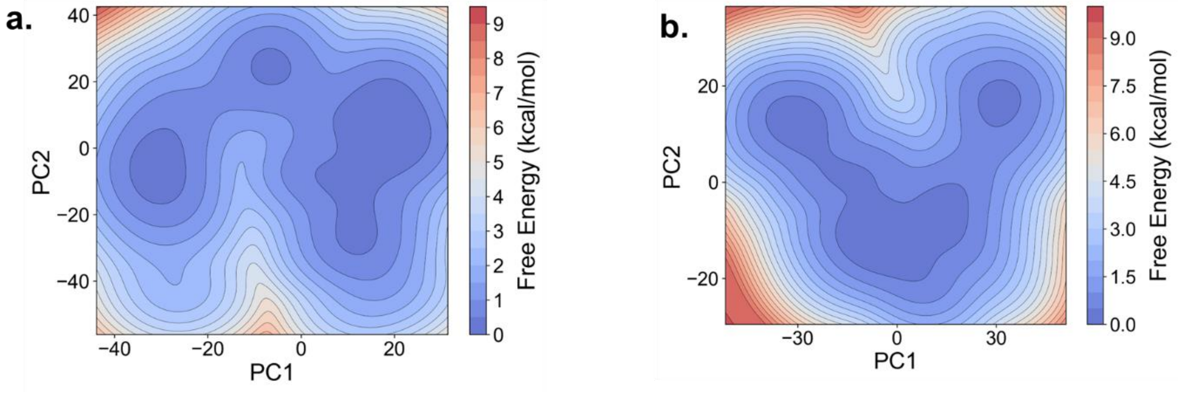

Supplementary Fig. 20. Free energy landscapes of the 2AcuB_1AcuC trimer in the apo and Ap4A-bound states.

A. Free energy landscape of the apo 2AcuB_1AcuC trimer projected onto the first two principal components (PCA1 and PCA2). **B.** Corresponding free energy landscape of the Ap4A-bound 2AcuB_1AcuC trimer. Both systems exhibit two major basins along the principal components, suggesting the presence of two dominant conformational states. However, the overall free energy surfaces are relatively flat, indicating low energy barriers and smooth transitions between these conformations. The similarity in the topography of the two landscapes suggests that Ap4A binding does not drastically alter the global conformational ensemble of the 2AcuB_1AcuC trimer but may subtly stabilize specific conformations relevant to inhibitory activity.

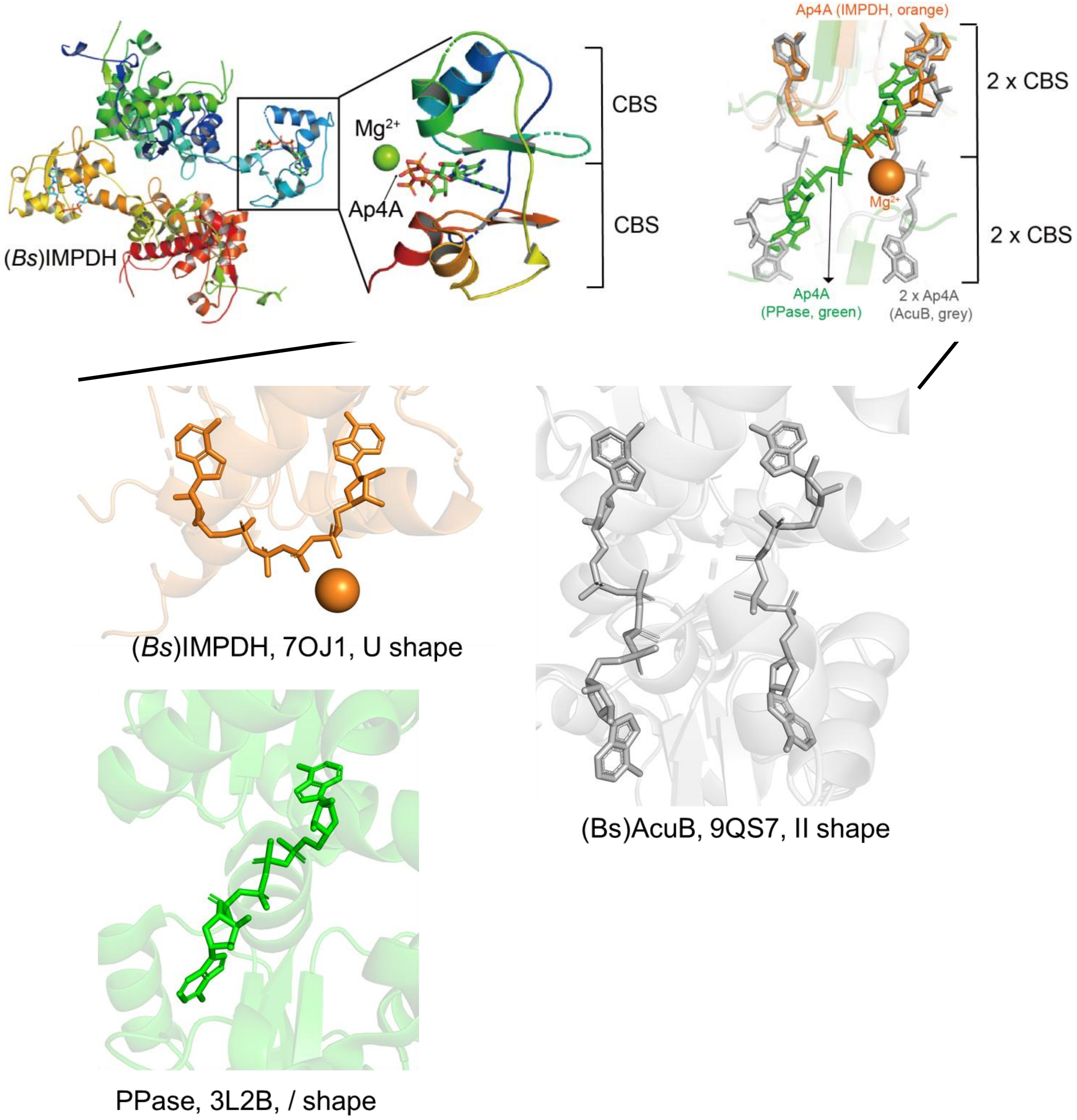

Supplementary Fig. 21. Binding mode of different CBS domains with Ap4A. Structure of (*Bs*)IMPDH, PDB ID: 7OJ1; pyrophosphatase (PPase), PDB ID: 3L2B; and (*Bs*)AcuB; PDB ID: 9QS7 (this study) are used.

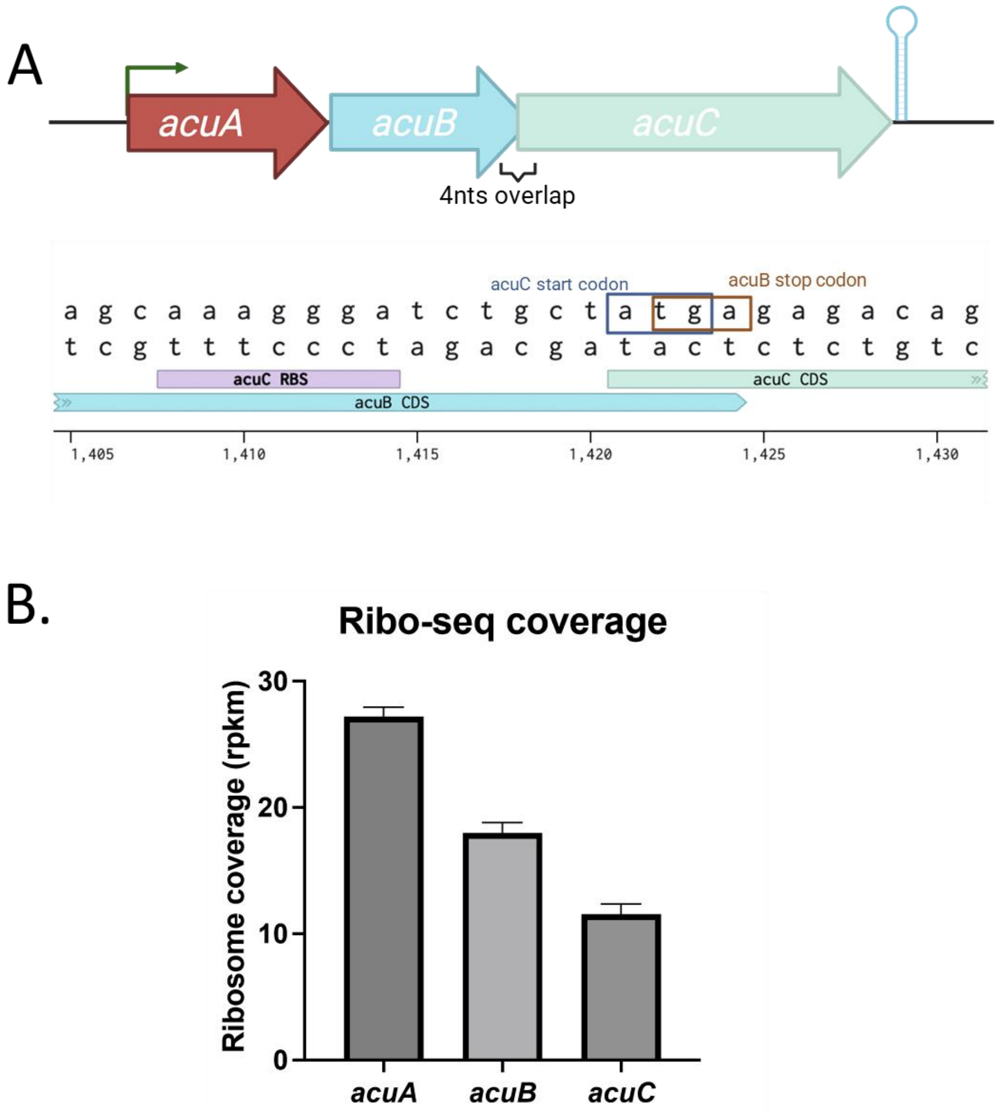

Supplementary Fig. 22. A. Topology of the *acuABC* operon and the overlapping open reading frames of *acuB* and *acuC*. **B**. Ribosome profiling (Ribo-seq) coverage of *acuA*, *acuB*, and *acuC*, shown as reads per kilobase of transcript per million mapped reads (RPKM). The analysis was performed using previously published Ribo-seq data. The dataset is available in the Gene Expression Omnibus under accession number GSE95211[6]. Source data are provided as a Source Data file

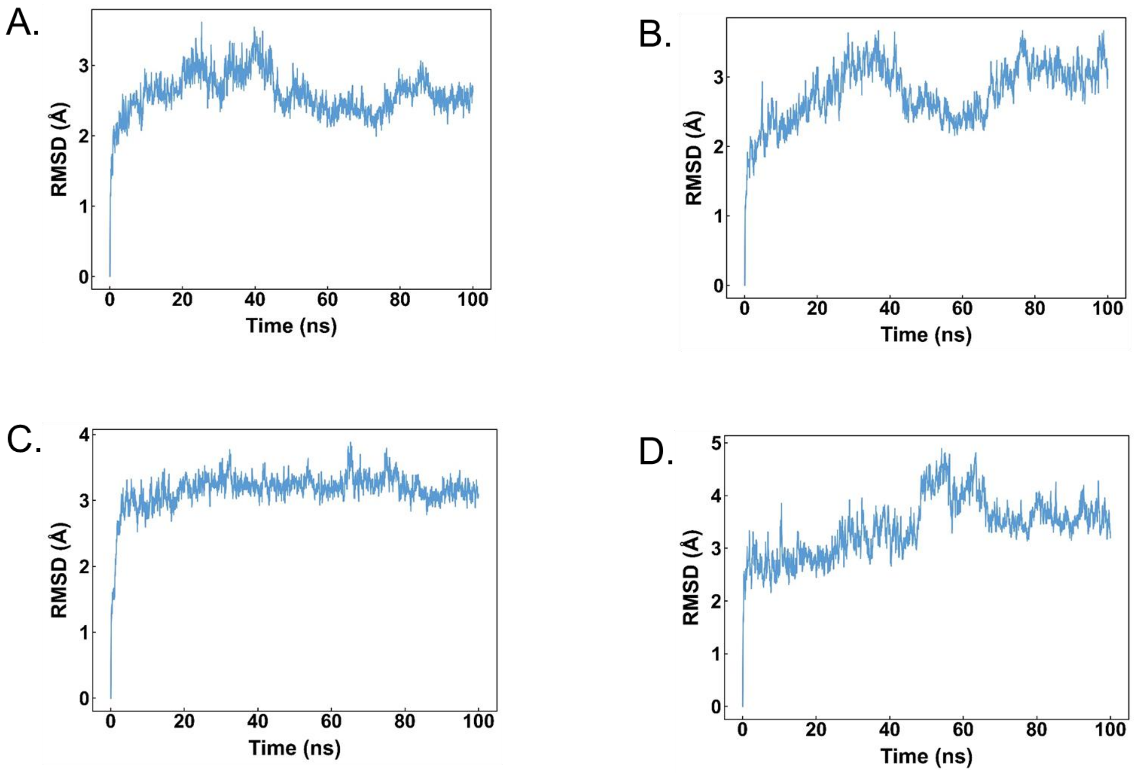

Supplementary Fig. 23. Evidence of system equilibration for molecular dynamics simulations. Root mean square deviation (RMSD) of Cα atoms during MD simulations. **A** and **B**, Time evolution of the backbone RMSD for the AcuB dimer in the apo state and in the Ap4A-bound state, respectively. **C** and **D**, Time evolution of the backbone RMSD for the 2AcuB–1AcuC trimer in the apo state and in the Ap4A-bound state, respectively.

Supplementary Table 1. Statistics of data collection, structural refinement and model validation.

	(*Bs*)AcuB bound to Ap4A
**Data collection**	
Space group	$P6_522$
**Cell dimensions**	
a, *b*, *c* (Å)	81.546
	81.546
	338.549
α, *β*, *γ* (°)	90.0
	90.0
	120.0
Wavelength (Å)	0.97626
Resolution (Å)	59.86 - 2.411 (2.497 - 2.411)
R_{merge}	0.06657 (1.555)
$I/\sigma I$	35.04 (2.58)
Completeness (%)	99.95 (100.00)
Redundancy	35 (40.1)
CC1/2	0.999 (0.879)
**Refinement**	
Resolution (Å)	59.86 - 2.411
No. reflections	26889 (2586)
R_{work} / R_{free}	0.2400 / 0.2834
No. atoms	3304
Protein	3196
Ligand/ion	106
Water	2
B-factors	99.89
Protein	100.04
Ligand/ion	77.30
Water	98.83
**R.m.s. deviations**	
Bond lengths (Å)	0.003
Bond angles (°)	0.57
**Ramachandran**	
Favoured (%)	97.47
Allowed (%)	2.53
Outliers (%)	0.00

Data were collected on P13 (DESY, Hamburg, Germany).
Values in parentheses are for the highest-resolution shell.

Supplementary Table 2. Overview of data obtained by hydrogen/deuterium exchange mass spectrometry (HDX-MS)

Protein	AcuB	AcuC
Conditions of H/D exchange	25 °C in 20 mM Tris-Cl pH 7.4, 150 mM NaCl, 20 mM $MgSO_4$ final D_2O = 90%	
Time course of H/D exchange	10/30/100/1,000/10,000 s	
Samples	1) AcuB<br>2) AcuB + Ap4A<br>3) AcuB/AcuC<br>4) AcuB/AcuC + Ap4A	1) AcuC<br>2) AcuC + Ap4A<br>3) AcuB/AcuC<br>4) AcuB/AcuC + Ap4A
Replicates	3 technical replicates (separate H/D exchange reactions)	
Number of Peptides	93	84
Average peptide length (aa)	13.14	12.23
Sequence coverage (%)	93.2	93.5
Redundancy	6.3	3.17
Back-exchange	No correction for back-exchange based on a fully deuterated sample conducted.	
Repeatability (average SD)	0.043 Da / 0.394%	0.060 Da / 0.540%
Significance criterium applied	5% difference in relative and 0.5 Da difference in absolute HDX[5]	

Supplementary Table 3. Plasmids used in this study.

Plasmids	Description
pLZ16	pET-24d-NHis-YabA, a 360 bp fragment of *yabA* from gDNA of *B. subtilis* with BsaI inserted in pET-24d
pLZ24	pET-24d-NStrep-AcuB-CHis, a 633 bp fragment of *acuB* from gDNA of *Geobacillus thermodenitrificans NG80-2*, with BsaI inserted in pET-24d
pLZ39	pLIKErep-AcuB-Cstrep, a 633 bp fragment of *acuB* from gDNA of *B. subtilis* for overexpression in *B. subtilis*
pLZ71	pET-24 -AcuB-CHis, R33E of *acuB* from pLZ24
pLZ73	pET-24 -AcuB-CHis, R51E of *acuB* from pLZ24
pLZ75	pET-24 -AcuB-CHis, D122A of *acuB* from pLZ24
pLZ96	pET-24d-NHis-AcuC, a 1164 bp fragment of *acuC* from gDNA of *Geobacillus thermodenitrificans NG80-2*, with BsaI inserted in pET-24d
pLZ130	pET-24d-NStrep-AcuB-CHis , R33E of *acuB* from pLZ24
pLZ144	pET-24d-NGST-*Gt*AcuB_ACT, ACT domain of *acuB* from gDNA of *Geobacillus thermodenitrificans NG80-2*, with BsaI inserted in pET-24d
pLZ223	pET-24d-NHis-TufA, a 1191 bp fragment of *TufA* from gDNA of *B. subtilis* with BsaI inserted in pET-24d
pLZ224	pET-24d-NHis-MurC a 1299 bp fragment of *MurC* from gDNA of *B. subtilis* with BsaI inserted in pET-24d
pLZ279	pET-24d-NStrep-*Gt*AcuB-CHis , M188A of *acuB* from pLZ24
pLZ280	pET-24d-NStrep-*Gt*AcuB-CHis , D89K of *acuB* from pLZ24
pLZ281	pET-24d-NStrep-*Gt*AcuB-CHis , E92R of *acuB* from pLZ24
pLZ282	pET-24 -*Gt*AcuC-NHis, R198A of *acuC* from pLZ96
pLZ283	pET-24 -*Gt*AcuC-NHis, R197E of *acuC* from pLZ96
pLZ284	pET-24 -*Gt*AcuC-NHis, R362E of *acuC* from pLZ96

Supplementary Table 4. Primers used in this study.

The nucleotide substitutions for each point mutation are highlighted and underlined in red.

Primers	Sequence 5'-3'
pLZ16-F	TTAAGGTCTCCCATGGGCCATCACCATCACCATCACGATAAAAAAGAGTTATTTGAT
pLZ16-R	TTAAGGTCTCCTCGAGTTATTTTTTATTTAAGAATGACAG
pLZ24-F	TTAAGGTCTCCCATGGGCATTGTTGAACAAGTCATGAAAACA
pLZ24-R	TTAAGGTCTCCTCGAGTGATGTAACCCCCGGCAAGT
pLZ39-F	TTAATCTAGATGATTGTTGAGCAAATCATGAAAAGAG
pLZ39-R	TTAAGCATGCTTATTTTTCAAATTGCGGATGGCTCCATGCGCTTAGCAGATCCCTTTGCTCTG
pLZ71-F	GAATTCCATATCGAGCATCTGCCT
pLZ71-R	AGGCAGATGCTCGATATGGAATTC
pLZ73-F	ACAGACGAGGACATGAAACAG
pLZ73-R	CTGTTTCATGTCCTCGTCTGTAATCATGCCGA
pLZ75-F	AAAAACCGCTTTGCTGCGGAC
pLZ75-R	GTCCGCAGCAAAGCGGTTTTT
pLZ96-F	TTAAGGTCTCCCATGGGCAGCGACAAGTGCGTATTTGTT
pLZ96-R	TTAAGGTCTCCTCGAGAGTGCCCGCGCGCCGCTGGCT
pLZ130-F	ATCGCATCGAACATCTCCCGGT
pLZ130-R	ACCGGGAGATGTTCGATGCGAT
pLZ144-F	TTAAGGTCTCCCATGGGCCAGCCGGGCTCGCAAAT
pLZ144-R	TTAAGGTCTCCTCGAGTAGCAGATCCCTTTGCTCTGACGG
pLZ223-F	TTAAGGTCTCCCATGGGCGCTAAAGAAAAATTCGACCGTTC
pLZ223-R	TTAAGGTCTCCTCGAGCTCAGTGATTGTAGAAACAACGC
pLZ224-F	TTAAGGTCTCCCATGGGCACTGTTTATCATTTTGTTGG
pLZ224-R	TTAAGGTCTCCTCGAGTGCCATGACGTTTTCGTAGG
pLZ279-F	AAACGGCGAACCCGATCAACTT
pLZ279-R	AAGTTGATCGGGTTCGCCGTTT
pLZ280-F	ACCCGCTCAAGTTCGTCGAAGAA
pLZ280-R	TTCTTCGACGAACTTGAGCGGGT
pLZ281-F	TCGATTTCGTCCGCGAAGTCGC
pLZ281-R	GCGACTTCGCGGACGAAATCGA
pLZ282-F	AGACAGGCCGCGCCTTGTTTC
pLZ282-R	GAAACAAGGCGCGGCCTGTCT
pLZ283-F	ATGAGACAGGCGAATACTTGTTTC
pLZ283-R	GAAACAAGTATTCGCCTGTCTCAT
pLZ284-F	ACCGATCCCAGAAAAAGCGGAAATTAG
pLZ284-R	CTAATTTCCGCTTTTTCTGGGATCGGT

Supplementary Table 5. Summary Table of Molecular Dynamics simulations

System	Box dimensions (Å, $\alpha=\beta=\gamma=$°)	Total atoms	Water molecules	Salt (Na^+)	Temperature	Simulation time	Notes
AcuB Dimer apo (Fig. 5d_apo)	100.914 × 91.590 × 115.567, 90 × 90 × 90	90,710	87,350	8	300 K	200 ns	aMD; generated using SWISS-MODEL, Ap4A docking by LeDock
AcuB Dimer Ap4A-bound (Fig. 5d_com)	100.100 × 92.102 × 115.959, 90 × 90 × 90	89,756	86,282	16	300 K	200 ns	aMD; SWISS-MODEL & Ap4A docking
AcuB2-AcuC1 Trimer (Fig. 5f_apo)	121.204 × 115.556 × 115.959, 90 × 90 × 90	139,614	133,142	21	450 K	200 ns	MD; trimer modeled by AlphaFold3; Zn^{2+} parameterized by MCPB.py
AcuB2-AcuC1 Trimer Ap4A-bound (Fig. 5f_com)	121.204 × 115.556 × 115.959, 90 × 90 × 90	139,713	133,127	29	450 K	200 ns	MD; AlphaFold3 model; RESP charges for Ap4A

Supplementary Table 6. Checklist Table of Molecular Dynamics simulations

**Reliability and reproducibility checklist for molecular dynamics simulations**<br>***All boxes must be marked YES by acceptance unless "Response not needed if No".**	**Yes**	**No**	**Response**<br>**(Please state where this information can be found in the text)**
**1. Convergence of simulations and analysis**			
1a. Is an evaluation presented in the text to show that the property being measured has equilibrated in the simulations<br>(*e.g.* time-course analysis)?	☒	☐	Method section for MD.
1b. Then, is it described in the text how simulations are split into equilibration and production runs and how much data were analyzed from production runs?	☒	☐	Method section for MD
1c. Are there at least 3 simulations per simulation condition with statistical analysis?	☒	☐	Caption of Figure 5
1d. Is evidence provided in the text that the simulation results presented are independent of initial configuration?	☒	☐	Method section for MD
**2. Connection to experiments**			
2a. Are calculations provided that can connect to experiments (*e.g.* loss or gain in function from mutagenesis, binding assays, NMR chemical shifts, J-couplings, SAXS curves, interaction distances or FRET distances, structure factors, diffusion coefficients, bulk modulus and other mechanical properties, *etc.*)?	☒	☐	Results section: Mechanistic insight into mediated AcuB inhibition and the effect of Ap4A
**3. Method choice**			
3a. Do simulations contain membranes, membrane proteins, intrinsically disordered proteins, glycans, nucleic acids, polymers, or cryptic ligand binding?	☒	☐	Method section for MD
3b. Is it described in the text whether the accuracy of the chosen model(s) is sufficient to address the question(s) under investigation (e.g. all-atom vs. coarse-grained models, fixed charge vs. polarizable force fields, implicit vs. explicit solvent or membrane, force field and water model, etc.)?	☒	☐	Method section for MD and Supplementary Figure 19
3c. Is the timescale of the event(s) under investigation beyond the brute-force MD simulation timescale in this study that enhanced sampling methods are needed?	☒	☐	Method section for MD and Supplementary Figures 19 and 23
If **YES**, are the parameters and convergence criteria for the enhanced sampling method clearly stated?	☒	☐	Supplementary Table 5
If **NO,** is the evidence provided in the text?	☐	☐	
**4. Code and reproducibility**			
4a. Is a table provided describing the system setup that includes simulation box dimensions, total number of atoms, total number of water molecules, salt concentration, lipid composition (number of molecules and type)?	☒	☐	Supplementary Table 5
4b. Is it described in the text what simulation and analysis software and which versions are used?	☒	☐	Method section for MD

4c. Are other parameters for the system setup described in the text, such as protonation state, type of structural restraints if applied, nonbonded cutoff, thermostat and barostat, etc.?	☐	☒	We did not take this into consideration
4d. Are initial coordinate and simulation input files and a coordinate file of the final output provided as supplementary files or in a public repository?	☒	☐	Supplementary Dataset 4
4e. Is there custom code or custom force field parameters?	☐	☒	Response not needed if **No**
If **YES**, are they provided as supplementary files or in a public repository?	☐	☐	

Supplementary References

1 Nakabayashi, M. *et al.* Crystal structure of a hypothetical protein, TTHA0829 from Thermus thermophilus HB8, composed of cystathionine-β-synthase (CBS) and aspartate-kinase chorismate-mutase tyrA (ACT) domains. *Extremophiles* **20**, 275-282 (2016).

2 Jumper, J. *et al.* Highly accurate protein structure prediction with AlphaFold. *Nature* **596**, 583-589 (2021).

3 Lammers, M. Post-translational Lysine Ac (et) ylation in Bacteria: A Biochemical, Structural, and Synthetic Biological Perspective. *Frontiers in microbiology* **12** (2021).

4 Finnin, M. S. *et al.* Structures of a histone deacetylase homologue bound to the TSA and SAHA inhibitors. *Nature* **401**, 188-193, doi:10.1038/43710 (1999).

5 Houde, D., Berkowitz, S.A., and Engen, J.R. (2011). The utility of hydrogen/deuterium exchange mass spectrometry in biopharmaceutical comparability studies. *Journal of pharmaceutical sciences* **100**, 2071-2086.

6 Lalanne, J.-B., Taggart, J.C., Guo, M.S., Herzel, L., Schieler, A., and Li, G.-W. (2018). Evolutionary Convergence of Pathway-Specific Enzyme Expression Stoichiometry. *Cell* ***173***, 749-761.e38.
