# Supplementary material for: A protein adaptor mediating Ap4A-dependent control of protein acetylation
Source: Nat Commun. 2026 Feb 23;17:3089. doi: 10.1038/s41467-026-70006-0 (PMC13039410; doi:10.1038/s41467-026-70006-0)
Supplement: Supplementary file 2 — Description of Additional Supplementary File [file 41467_2026_70006_MOESM2_ESM.pdf]

The Description of Additional Supplementary Files

Supplementary Dataset 1: Summary of Pull-Down experiments using Strep-tag II-AcuB as a bait.

Supplementary Dataset 2: Summary of Pull-Down experiments using Strep-tag II as a bait.

Supplementary Dataset 3: Raw data from HDX experiments

Supplementary Dataset 4: Initial and final configurations of MD trajectories
